# Physics-Preserving Attention-Guided Artifact Removal for Spaceborne Optical Images

**DOI:** 10.3390/jimaging12090414

**Published:** 2026-09-03

**Authors:** Shuxiang Cai, Zuoxun Hou, Haian Zhou, Zheng Pan, Dong Wang

**Affiliations:** 1 Institute of Information Science, Beijing Jiaotong University, Beijing 100044, China; 24120273@bjtu.edu.cn; 2 Beijing Institute of Space Mechanics & Electricity, China Academy of Space Technology, Beijing 100094, China; hzx_007xjtu@163.com (Z.H.); zhouhaian@cast508.cn (H.Z.); panzheng@cast508.cn (Z.P.)

**Keywords:** spaceborne optical images, artifact removal, generative image editing, editing-induced radiometric drift, cross-attention masks, multi-scale fusion, radiometric fidelity

## Abstract

Artifacts, including halos and saturated bright spots, are common in spaceborne optical images and can degrade the reliability of star extraction, space object detection, and photometric analysis. Traditional signal-processing methods, such as morphological filtering and low-rank decomposition, rely on fixed priors that may fail under complex artifact morphologies. Deep restoration networks can improve visual quality but do not explicitly enforce radiometric consistency. Multimodal instruction-driven editing models provide semantic localization capability, but probabilistic diffusion resampling can introduce uncontrolled pixel changes in non-target regions, compromising pixel-level physical consistency. We refer to this problem as *editing-induced radiometric drift*. To address this problem, we propose PARE, a physics-preserving attention-guided artifact removal framework for spaceborne optical images. Instead of directly using the edited image as the final restoration, PARE treats it as a candidate restoration and derives artifact-region constraints from the image-to-text cross-attention sub-block of the MM-DiT joint attention matrix. These constraints are combined with multi-scale fusion to restrict generative modification to localized artifact regions, thereby enabling artifact suppression while reducing unintended changes in non-target regions. Experiments on simulated and real spaceborne optical image datasets show that PARE achieves effective artifact suppression while improving radiometric preservation. On real on-orbit images, PARE reaches 92.95% artifact mean reduction (AMR) and 98.57% artifact energy reduction (AER), reduces the outer-region mean absolute error (O-MAE) to 0.54, and improves the outer-region structural similarity index (O-SSIM) to 0.997. It also yields the smallest background shifts among all compared methods. These results indicate that PARE provides a favorable trade-off between artifact suppression and radiometric fidelity and offers a practical way to apply generative models to scientific imaging tasks that require pixel-level physical consistency.

## 1. Introduction

Space-based optical sensors are essential for space object surveillance, spacecraft attitude determination, and deep-sky astronomical observations. The reliability of these applications depends critically on image fidelity because subtle pixel-level distortions can propagate into measurable errors in flux estimation, centroid localization, and faint-target detection [1,2]. In practice, spaceborne optical images are often affected by diverse artifacts, including stray-light-induced halos, saturated bright spots, cosmic-ray-induced transient bright pixels, and banding patterns caused by readout electronics nonuniformity or imperfect flat-field correction.

**Figure 1:**
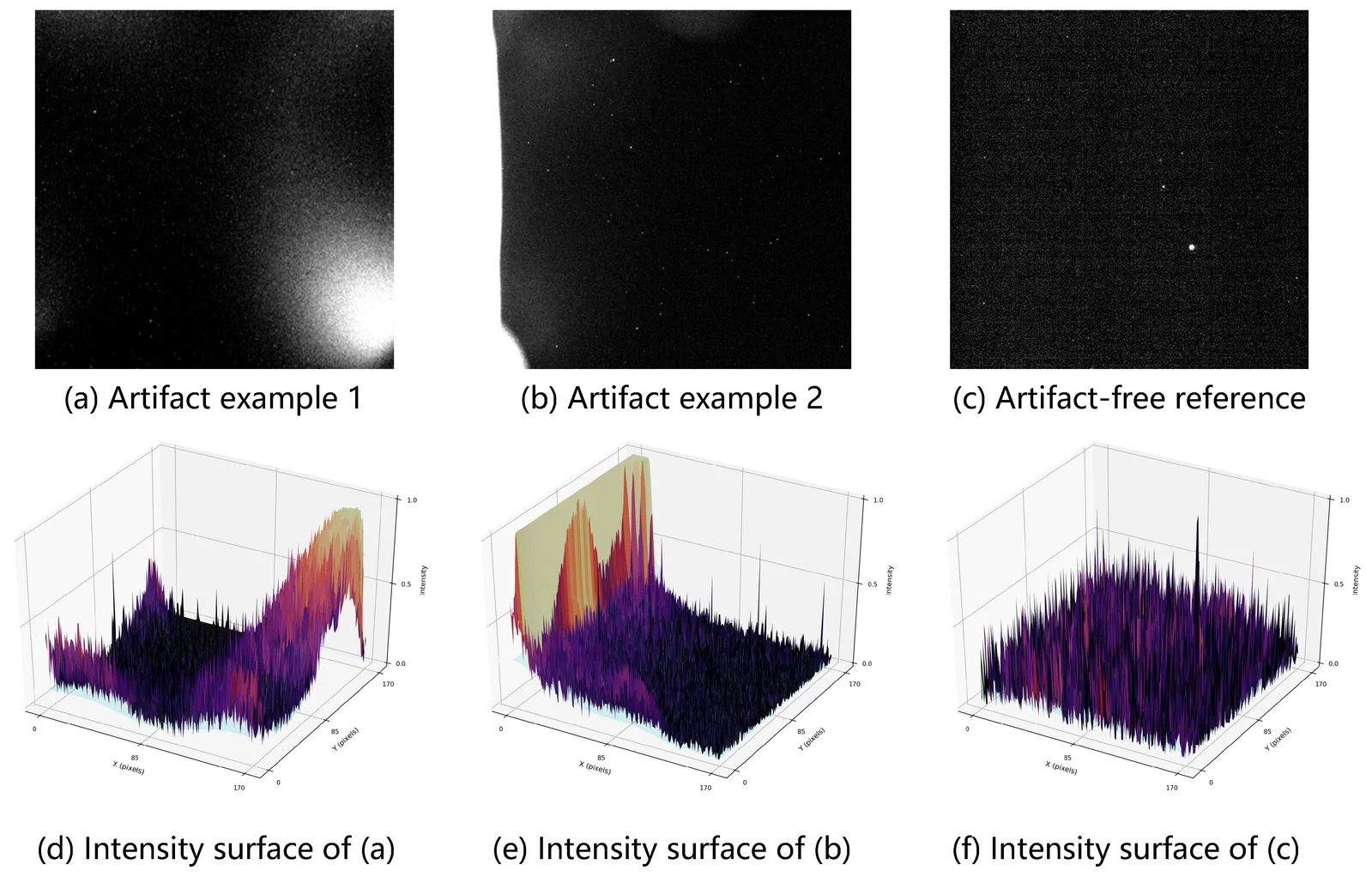
Structured artifacts and their intensity distributions. (**a**,**b**) Artifact-contaminated spaceborne optical images. (**c**) An artifact-free reference image. (**d**–**f**) Corresponding intensity surface plots. Compared with the artifact-free reference, artifact-contaminated images exhibit spatially coherent and smoothly varying intensity structures, indicating a structured degradation pattern rather than random noise.

These degradations differ from simple random noise in both spatial structure and radiometric effect. As shown in Figure 1, artifact-contaminated images contain organized intensity patterns, saturated regions, point-like anomalies, and large-area structural disturbances. These patterns are clearly different from sparse stellar responses and weak background fluctuations in artifact-free images.

**Limitations of existing methods.** Artifacts in spaceborne optical images are difficult to remove because of their structured and variable characteristics. Traditional signal-processing methods, such as the morphological top-hat transform [3], polynomial background fitting [4], and low-rank sparse decomposition [5], are interpretable but rely on fixed morphological priors or statistical assumptions. These assumptions may break down when artifacts have varying scales, soft boundaries, spatially coherent intensity patterns, or overlaps with stellar structures and faint targets. Data-driven restoration networks, such as DnCNN [6] and partial convolution inpainting [7], can improve visual quality, but they do not explicitly preserve radiometric fidelity and may introduce unintended changes in non-target regions.

**Figure 2:**
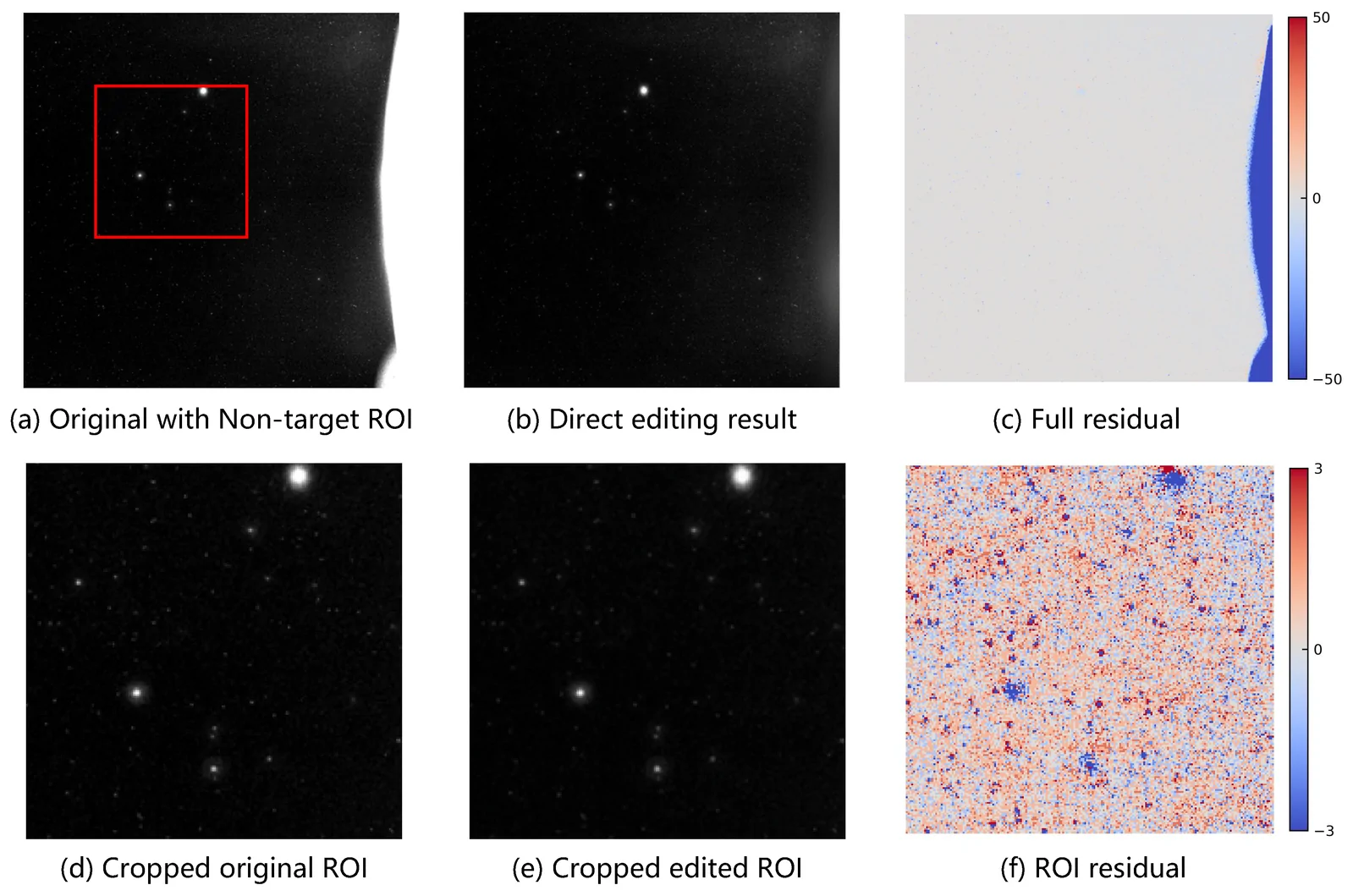
Illustration of editing-induced radiometric drift. (**a**) Original spaceborne optical image with a selected non-target ROI. (**b**) Direct diffusion-based editing result. (**c**) Full-image residual map. (**d**,**e**) Cropped original and edited ROIs. (**f**) Residual map within the selected ROI. The selected ROI lies outside the intended artifact editing region.

**Opportunity and challenge.** The visually recognizable patterns of spaceborne image artifacts make them suitable targets for instruction-driven multimodal generative editing [8,9]. Given a prompt such as “remove the artifacts while preserving stars”, a diffusion model can provide artifact localization cues through cross-attention maps [10,11], reducing the need for hand-crafted rules or pixel-level artifact annotations. However, the generated image should not be directly treated as a radiometrically faithful restoration result. As illustrated in Figure 2, direct diffusion-based editing can introduce radiometric changes, even in non-target regions outside the intended artifact editing area. Although such changes may be visually subtle, they are important for spaceborne optical images, where each pixel carries radiometric information. We refer to this issue as the *editing-induced radiometric drift* problem.

**Proposed approach.** To address this problem, we propose PARE, a physics-preserving attention-guided artifact removal framework for spaceborne optical images. Rather than treating the generated image as the final restoration, PARE uses the diffusion model primarily as a semantic prior for artifact localization. Specifically, cross-attention maps from the MM-DiT joint attention matrix [11] are aggregated and refined into an explicit artifact mask, while the original and edited images are decomposed using a multi-scale Laplacian pyramid [12]. The resulting image retains high-frequency stellar structures from the original image and replaces low-frequency background components only within the attention-derived artifact mask. By enforcing this spatial constraint, PARE confines generative modifications to artifact regions and reduces unintended radiometric changes outside the target mask.

**Contributions.** The main contributions of this work are summarized as follows:

1. We formulate the *editing-induced radiometric drift* problem for spaceborne optical images and show, through quantitative analysis, that direct generative editing can introduce substantial unintended pixel modifications in non-target regions, thereby compromising the radiometric integrity required for scientific measurements.
2. We propose an annotation-free attention-guided mask extraction strategy that converts I2T cross-attention responses from the MM-DiT double-stream blocks [11] into explicit artifact-region constraints without manual annotations.
3. We design a physics-preserving multi-scale fusion mechanism that preserves high-frequency stellar structures from the original image and uses edited low-frequency background components only within masked artifact regions, thereby reducing non-target radiometric drift.
4. We introduce a physics-motivated evaluation protocol that separately quantifies the effectiveness of artifact suppression and non-target-region radiometric fidelity. Using this protocol, we validate PARE on simulated and real spaceborne optical image datasets and demonstrate a favorable trade-off between artifact suppression and radiometric fidelity.

The remainder of this paper is organized as follows. Section 2 reviews related work. Section 3 presents the proposed PARE framework and experimental setup. Section 4 reports the experimental results. Section 5 discusses the findings and limitations. Section 6 concludes this paper.

## 2. Related Work

### 2.1. Artifact Suppression in Spaceborne Optical Images

Morphological top-hat filtering [3] can suppress brightness structures that are smaller than the structuring element, but it often leaves edge residuals and distorts stellar profiles when large-area diffuse artifacts exceed the filter scale. Polynomial surface fitting [4] and background estimation based on local statistics implicitly assume spatially stationary background variations, an assumption that is often violated by nonuniform stray-light contamination and other smoothly varying artifacts across the focal plane. Low-rank sparse decomposition [5] provides a principled formulation for separating mixed interference components, but large connected artifacts, saturated regions, and spatially coherent disturbances do not always match the assumed low-rank or sparse structures. As a result, these methods may produce incomplete artifact separation or incorrectly assign stellar signals to the artifact component.

Deep restoration networks can improve visual reconstruction but may introduce systematic photometric biases that are unsuitable for scientific imaging. DnCNN [6] shows that residual learning with batch normalization achieves strong denoising performance across multiple degradation types, yet its training objective does not guarantee radiometric fidelity. Partial convolution inpainting [7] handles irregular missing regions by conditioning convolution responses on valid pixels, but it optimizes natural-image appearance rather than physical measurement consistency. In summary, existing methods lack explicit constraints on physical measurement consistency, which is a critical limitation for scientific image analysis.

### 2.2. Multimodal Instruction-Driven Image Editing

SDEdit [13] enables controlled image synthesis and editing by adding structured noise to an input image and denoising it with a pretrained stochastic differential equation prior, without task-specific training. InstructPix2Pix [8] extends this direction by training a conditional diffusion model on a large dataset of instruction–image pairs synthesized from GPT-3 and Stable Diffusion, enabling image editing from natural language instructions at inference time without task-specific annotations. Subsequent methods improve spatial controllability. Blended Diffusion [12] restricts the editing extent by compositing original and generated content during the diffusion denoising trajectory, preserving unedited regions through progressive spatial blending. Prompt-to-Prompt [10] enables region-aware editing by injecting cross-attention maps from a source prompt into the generation process of an edited prompt, allowing localized semantic changes without explicit spatial masks. The more recent FLUX.1 Kontext [9] introduces a rectified-flow transformer that unifies image generation and editing through in-context sequence concatenation of text and image inputs, achieving strong preservation of object identity and character consistency across iterative editing turns.

Although these methods substantially improve visual quality, their fidelity constraints remain insufficient for the radiometric requirements of space-based imaging. Intensity changes that are perceptually negligible in natural photographs can become systematic errors in photometric and astrometric measurements when they appear in spaceborne optical images. This gap motivates the *editing-induced radiometric drift* problem addressed in this paper.

### 2.3. Attention Mechanisms for Spatial Control

Cross-attention maps between text tokens and image spatial positions encode the semantic focus of a diffusion model during instruction execution. Prompt-to-Prompt [10] shows that injecting these attention maps across generation steps preserves spatial layout and enables fine-grained semantic control, establishing cross-attention as a useful localization signal. DIFT [14] shows that intermediate activations extracted from a frozen diffusion network can serve as dense features for unsupervised semantic correspondence, indicating that spatially meaningful representations emerge inside diffusion architectures without task-specific supervision. Seg4Diff [11] further analyzes the MM-DiT joint attention structure and identifies intermediate semantic-grounding expert layers, whose I2T sub-block consistently aligns text tokens with spatially coherent image regions and produces high-quality zero-shot segmentation masks.

These works demonstrate that diffusion internals can provide effective spatial localization signals, but they mainly target visual quality in natural images rather than pixel-level physical fidelity in scientific data. PARE builds on this line of work by extracting I2T cross-attention responses from the MM-DiT double-stream blocks, following the analysis in [11], and refining them into an explicit binary artifact mask. This mask is coupled with frequency-aware fusion rules in a multi-scale Laplacian pyramid, converting attention from a soft generation guide into a hard spatial constraint so that non-target pixels are preserved from the observed sensor data.

## 3. Materials and Methods

**Figure 3:**
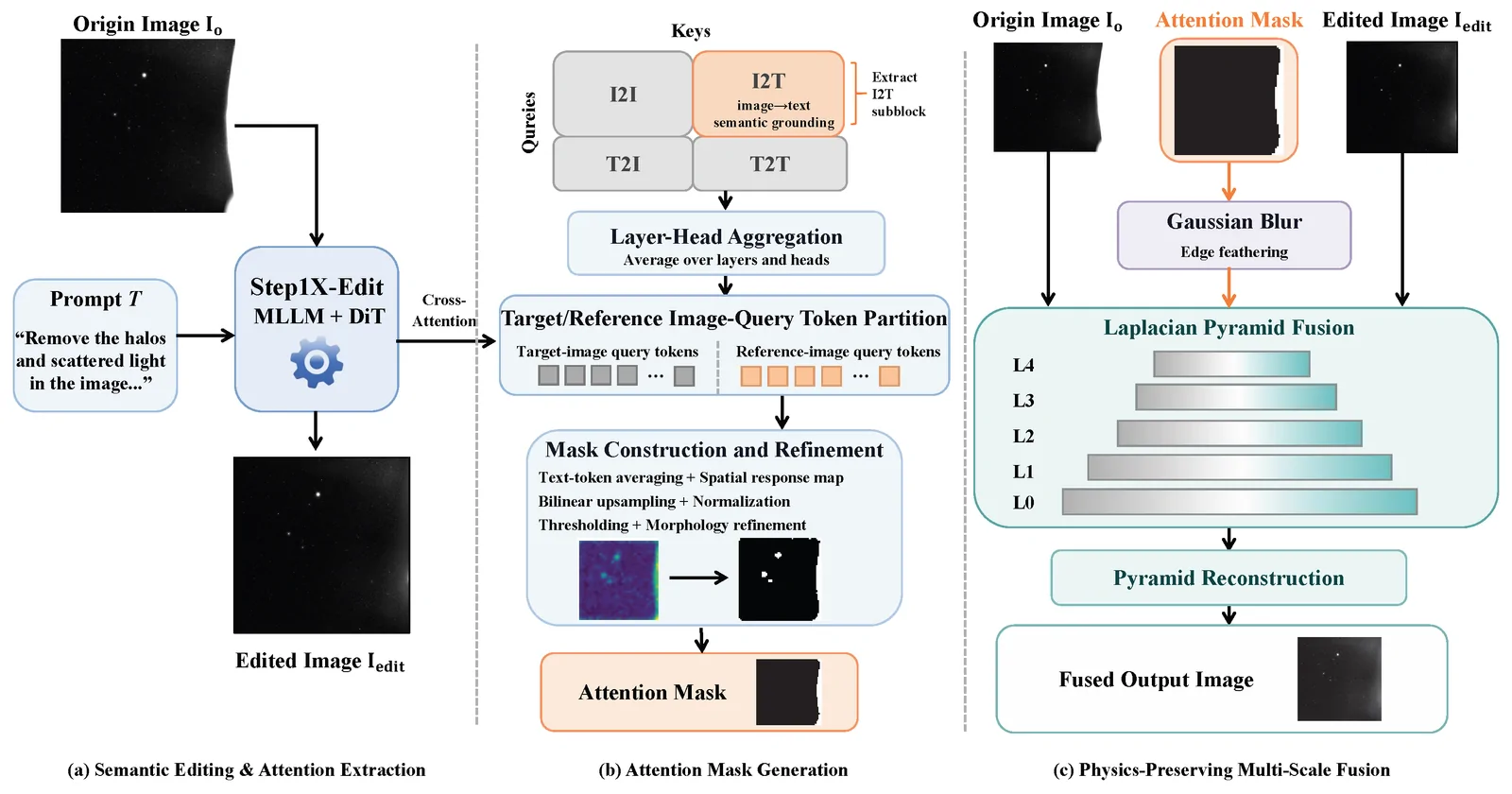
Overall architecture of the proposed PARE framework. (**a**) **Semantic editing and attention extraction:** An artifact-contaminated spaceborne optical image ($I_{o}$) and a text prompt (T) are fed into Step1X-Edit [15] (MLLM + DiT), which produces an edited candidate image ($I_{\mathrm{edit}}$) and exposes internal cross-attention maps for downstream mask generation. (**b**) **Attention mask generation:** The image-to-text (I2T) sub-block is extracted from the MM-DiT joint attention matrix following [11] and aggregated across layers and heads. Reference-token selection separates artifact-correlated tokens from background tokens. The resulting score map is then upsampled, thresholded, and refined by morphological operations to produce a binary attention mask that localizes the artifact region. (**c**) **Physics-preserving multi-scale fusion:** $I_{o}$, $I_{\mathrm{edit}}$, and the Gaussian-feathered attention mask are decomposed into a four-level Laplacian pyramid. At each level, edited low-frequency components inside the mask replace the corresponding artifact-contaminated background, while original high-frequency stellar structures are retained from $I_{o}$. Pyramid reconstruction produces the final fused output with constrained pixel modification outside the attention-derived mask.

The overall pipeline of PARE is illustrated in Figure 3. Given a spaceborne optical image ($I_{o}\in R^{H\times W}$) containing structured artifacts, the goal is to produce a clean and radiometrically faithful restoration ($\hat{I}$) through three stages: (i) multimodal editing and candidate image generation, (ii) cross-attention mask extraction and refinement, and (iii) multi-scale physics-preserving fusion.

### 3.1. Multimodal Editing and Candidate Image Generation

A pre-trained multimodal instruction-editing model (G), implemented with Step1X-Edit [15], is applied to the input image ($I_{o}$) under a natural language prompt (T, e.g., “Remove the structured artifacts around the stars while keeping the background dark and the stars sharp”):

(1) $$I_{\mathrm{edit}}=G\left(I_{o},T\right).$$

Architecturally, Step1X-Edit integrates an MLLM with a DiT backbone [16,17]. During inference, the input image and instruction are jointly processed to extract multimodal conditioning features, which guide the DiT in the diffusion denoising process to produce the edited candidate ($I_{\mathrm{edit}}$). Concurrently, the joint multimodal attention responses computed within the DiT are preserved for subsequent artifact localization.

Although the generated output ($I_{\mathrm{edit}}$) effectively suppresses most artifact-related structures, the probabilistic resampling inherent in the diffusion process may introduce unintended radiometric deviations. Consequently, $I_{\mathrm{edit}}$ is used solely as a candidate restoration source for downstream fusion rather than as the final restoration result.

### 3.2. Cross-Attention Mask Extraction and Refinement

Seg4Diff [11] decomposes the joint multimodal attention of MM-DiT into I2I, I2T, T2I, and T2T interactions and shows that I2T attention is particularly informative for image–text semantic grounding. Motivated by this observation, we extract I2T attention maps from Step1X-Edit to localize structured artifacts.

We extract the attention responses at the denoising step ($t^{*}=8$) within the 28-step sampling trajectory. Seg4Diff reports that the attention logits at this step exhibit clear semantic grouping and achieve the best segmentation performance among the evaluated timesteps. Based on these findings, we fix $t^{*}=8$ as the attention-extraction step for all images, without image-specific adjustment.

Under classifier-free guidance, we retain the conditional branch associated with the natural-language prompt. At $t^{*}$, the joint attention matrix of each retained double-stream block (*b*) and attention head (*h*) is partitioned into I2I, I2T, T2I, and T2T sub-blocks, from which the I2T sub-block is extracted. We denote the extracted sub-block as $A_{I2T}^{\left(b,h\right)}\;\in \;R^{L_{\mathrm{img}}\times L_{\mathrm{txt}}}$, where image tokens act as queries and text tokens act as keys.

The I2T responses are aggregated over all retained double-stream blocks and attention heads:

(2) $${\bar{A}}_{I2T}=\frac{1}{N_{b}N_{h}}\sum_{b=1}^{N_{b}}\sum_{h=1}^{N_{h}}A_{I2T}^{\left(b,h\right)},\;\;\;\;{\bar{A}}_{I2T}\in R^{L_{\mathrm{img}}\times L_{\mathrm{txt}}},$$

where $N_{b}$ and $N_{h}$ denote the numbers of retained double-stream blocks and attention heads, respectively.

Based on the fixed token-packing order of the underlying model, we extract the rows corresponding to the reference-image query tokens (indexed by $Q_{\mathrm{ref}}$) from the I2T attention matrix. To avoid manual selection of specific artifact keywords and mitigate the risk of biased segmentation, we uniformly average the cross-attention across all text tokens on the extracted reference-image query. This yields a robust and comprehensive spatial prior, computed as follows:

(3) $$\begin{array}{cc} {\bar{A}}_{\mathrm{ref}}\; & ={\bar{A}}_{I2T}\left[Q_{\mathrm{ref}},:\right],\\ r_{\mathrm{ref}}\; & =\frac{1}{L_{\mathrm{txt}}}\sum_{v=1}^{L_{\mathrm{txt}}}{\bar{A}}_{\mathrm{ref}}\left[:,v\right]. \end{array}$$

The reference-image response is reshaped according to its spatial token arrangement and bilinearly upsampled to the image resolution:

(4) $$R=\mathrm{Upsample}\left(\mathrm{Reshape}\left(r_{\mathrm{ref}}\right);H,W\right).$$

The spatial response is min–max-normalized to standardize the numerical range of the stored heatmap:

(5) $$\tilde{R}\left(x,y\right)=\frac{R(x,y)-\min(R)}{\max(R)-\min(R)+\epsilon },$$

where ϵ is a small constant for numerical stability and $\tilde{R}\in {\left[0,1\right]}^{H\times W}$. This normalization is used to obtain a consistent heatmap representation. The subsequent mask extraction is based on the percentile ranking of the responses rather than on a fixed absolute intensity threshold.

Before thresholding, the normalized heatmap is spatially smoothed using a 5×5 Gaussian kernel:

(6) $$S={\mathrm{GaussBlur}}_{5\times 5}\left(\tilde{R}\right).$$

We retain the pixels with the highest responses using a fixed percentile ratio. Let *p* denote the retained proportion, and let $Q_{1-p}\left(S\right)$ denote the (1−p)-quantile of the values in S. The percentile threshold ($\tau_{p}$) and the initial binary mask are defined as

(7) $$\tau_{p}=Q_{1-p}\left(S\right),\;\;\;\;M_{0}\left(x,y\right)=I\left[S\left(x,y\right)>\tau_{p}\right].$$

We fix p=0.30 for all images such that pixels within the highest 30% of the smoothed response distribution are retained. Here, p=0.30 denotes the retained percentile proportion rather than an absolute response threshold. The numerical value of $\tau_{p}$ is automatically determined from the response distribution of each image.

The initial binary mask is refined by morphological closing and hole filling to connect fragmented regions and remove interior holes, followed by a standard morphological dilation to ensure complete coverage of the artifact regions:

(8) $$M={\mathrm{Dilate}}_{3\times 3}\left(\mathrm{FillHoles}\left({\mathrm{Closing}}_{5\times 5}\left(M_{0}\right)\right)\right),$$

where $M\in {\left\{0,1\right\}}^{H\times W}$ is the final artifact-region mask. Mask values of one indicate regions in which the edited candidate contributes during subsequent fusion, whereas values of zero indicate regions dominated by the original observation.

The attention-extraction step, retained block range, aggregation rule, retained percentile ratio, and mask-refinement settings are fixed throughout the dataset. The procedure therefore requires neither image-specific parameter tuning, pixel-level annotations, nor auxiliary segmentation networks.

### 3.3. Multi-Scale Physics-Preserving Fusion

Given the original image ($I_{o}$), the edited candidate ($I_{\mathrm{edit}}$), and the artifact-region mask (M), PARE performs spatially constrained multiband fusion. The objective is to introduce artifact-suppressed information from $I_{\mathrm{edit}}$ within the localized artifact region while retaining the measured radiometric and structural information of the original observation in regions with low mask responses. In this context, physics preservation refers to constraint of the generative modification with the original observation rather than directly using the diffusion-edited image as the restoration result.

#### 3.3.1. Laplacian Pyramid Decomposition

Gaussian and Laplacian pyramids are constructed for both the original and edited images. Let s∈{o,e} denote the image source, where $I_{o}=I_{o}$ and $I_{e}=I_{\mathrm{edit}}$. The Gaussian and Laplacian components are defined as

(9) $$\begin{array}{cc} G_{s}^{0} & =I_{s},\;\;\;\;\;G_{s}^{k+1}=\mathrm{PyrDown}\left(G_{s}^{k}\right),\\ L_{s}^{k}\; & =G_{s}^{k}-\mathrm{PyrUp}\left(G_{s}^{k+1}\right),\;\;\;k=0,\ldots ,K-1,\\ L_{s}^{K} & =G_{s}^{K}, \end{array}$$

where *K* denotes the number of pyramid reductions. We use K=4, which produces four detail components and one coarsest low-frequency residual. This decomposition enables artifact correction across multiple spatial-frequency bands without directly replacing the complete image.

#### 3.3.2. Scale-Adaptive Mask-Guided Fusion

The binary artifact mask (M) is first Gaussian-smoothed to convert its hard boundary into continuous fusion weights. A multi-scale mask sequence is then generated through progressive downsampling:

(10) $$W^{0}=\mathrm{clip}\left(G_{\sigma_{M}}*M,0,1\right),\;\;\;W^{j+1}=\mathrm{PyrDown}\left(W^{j}\right),\;\;\;j=0,\ldots ,K-1,$$

where $G_{\sigma_{M}}$ denotes a Gaussian kernel with smoothing scale $\sigma_{M}$. For each Laplacian component, the associated mask representation is spatially aligned with the resolution of that component. Let ${\tilde{M}}^{k}\in {\left[0,1\right]}^{H_{k}\times W_{k}}$ denote the resulting fusion weight for the *k*-th component.

The original and edited representations are blended at every pyramid level:  

(11) $$L_{f}^{k}=\left(1-{\tilde{M}}^{k}\right)⊙L_{o}^{k}+{\tilde{M}}^{k}⊙L_{e}^{k},\;\;\;\;\;k=0,\ldots ,K,$$

where ⊙ denotes element-wise multiplication. A small value of ${\tilde{M}}^{k}$ preserves the corresponding component of the original observation, whereas a large value introduces a stronger contribution from the edited candidate. Intermediate values provide smooth spatial transitions between the two sources. Consequently, the edited candidate contributes across multiple frequency bands, while its influence remains spatially constrained by the artifact-derived mask.

#### 3.3.3. Pyramid Reconstruction

The fused image is recovered through inverse Laplacian pyramid reconstruction. Starting from the coarsest residual, the reconstruction proceeds recursively as

(12) $$\begin{array}{cc} Y^{K}\; & =L_{f}^{K},\\ Y^{k}\; & =L_{f}^{k}+\mathrm{PyrUp}\left(Y^{k+1}\right),\;\;\;k=K-1,\ldots ,0,\\ \hat{I}\; & =\mathrm{clip}\left(Y^{0}\right), \end{array}$$

where clip(·) constrains the reconstructed image to the valid intensity range.

This multiband fusion strategy preserves observation-dominant content in regions with low fusion weights and introduces edited information within localized artifact regions. Gaussian mask smoothing and multi-scale fusion further reduce visible seams and abrupt radiometric transitions around artifact boundaries.

The overall algorithmic workflow is summarized in Algorithm 1. Starting from the input contaminated image, PARE sequentially performs multimodal generative editing, cross-attention artifact mask localization, and constrained multi-scale Laplacian fusion to achieve physics-consistent artifact suppression.
**Algorithm 1**
PARE: Physics-Preserving Artifact Removal for Spaceborne Optical Images
**Require:** Artifact-contaminated image $I_{o}\in R^{H\times W}$, text prompt T**Require:** Fixed hyperparameters: denoising step $t^{*}=8$, percentile p=0.30, pyramid levels K=4**Ensure:** Restored artifact-suppressed image $\hat{I}$**Stage 1: Multimodal Editing & Attention Extraction**  1:$I_{\mathrm{edit}}\leftarrow G\left(I_{o},T\right)$  2:Collect intermediate I2T attention maps $\left\{A_{I2T}^{\left(b,h\right)}\right\}$ at denoising step $t^{*}$ during inference**Stage 2: Cross-Attention Mask Generation**  3:Aggregate I2T attention and extract reference response $r_{\mathrm{ref}}$ (Equations (2) and (3))  4:Upsample and min-max normalize to obtain spatial heatmap $\tilde{R}$ (Equations (4) and (5))  5:Apply Gaussian smoothing: $S\leftarrow {\mathrm{GaussBlur}}_{5\times 5}\left(\tilde{R}\right)$  6:Compute percentile threshold: $\tau_{p}=Q_{1-p}\left(S\right)$  7:Generate initial binary mask: $M_{0}\left(x,y\right)\leftarrow I\left[S\left(x,y\right)>\tau_{p}\right]$  8:Refine mask via morphology: $M\leftarrow \mathrm{Dilate}(\mathrm{FillHoles}\left({\mathrm{Closing}}_{5\times 5}\left(M_{0}\right)\right))$**Stage 3: Multi-Scale Physics-Preserving Fusion**  9:Construct Laplacian pyramids $\left\{L_{o}^{k}\right\}$ and $\left\{L_{e}^{k}\right\}$ for $I_{o}$ and $I_{\mathrm{edit}}$10:Generate multi-scale smoothed fusion weights $\left\{{\tilde{M}}^{k}\right\}$ from M (Equation (10))11:**for** k=0 **to** *K* **do**12:   $L_{f}^{k}\leftarrow \left(1-{\tilde{M}}^{k}\right)⊙L_{o}^{k}+{\tilde{M}}^{k}⊙L_{e}^{k}$13:**end for**14:Reconstruct final image $\hat{I}$ from fused pyramid $\left\{L_{f}^{k}\right\}$ (Equation(12)) 15:**return** $\hat{I}$

### 3.4. Experimental Setup

#### 3.4.1. Datasets

We employ a simulated dataset and a real spaceborne optical image dataset for model fine-tuning and evaluation.

The simulated dataset comprises 1500 images randomly sampled from a collection of 7320 pristine, noise-free Gaia star maps. These images are resized to 512×512 pixels and normalized to [0,1]. To simulate realistic sensor environments, both Gaussian noise (σ∈[0.1,0.3]) and salt-and-pepper noise (p∈[0.008,0.01]) are simultaneously injected to produce artifact-free noisy references. Subsequently, synthetic artifact patterns are superimposed. For each image, two to three artifact instances are randomly generated as either strip artifacts (length: 512 pixels, width ∈[20,25] pixels) or circular artifacts (radius ∈[20,80] pixels, Gaussian decay σ=r/2.5). These instances are fused into a single artifact map via element-wise maximization. A global intensity factor (∈[0.6,1.8]) is then sampled to scale the artifact map, which is additively blended with the noisy reference, clipped to [0,1], and saved as an 8-bit grayscale image. Concurrently, a binary mask corresponding to the artifact map is generated. To prevent data leakage, these 1500 pairs are strictly partitioned into 1000 pairs for training and fine-tuning, a validation set of 200 pairs, and an independent test set of 300 pairs for evaluation. The splitting is performed randomly at the image level to ensure no scene overlap.

The real dataset contains 103 on-orbit images (1024×1024, 8-bit RGB) acquired by a space-based surveillance sensor, featuring natural artifacts like halos, saturated spots, and scattered light. Since artifact-free references are physically unavailable, this dataset is strictly reserved for no-reference evaluation, with no images used for training or parameter selection.

#### 3.4.2. Baselines

We compare PARE with five representative baselines covering traditional filtering, supervised restoration, and instruction-guided image editing. TopHat [3] is used as a traditional morphology-based baseline for artifact and background correction and does not require training. DnCNN [6] is used as a fully supervised restoration baseline. For instruction-guided image editing, we compare PARE with InstructPix2Pix [8], FLUX.1-Kontext-dev [9], and LongCat-Image-Edit [18].

To ensure a fair and reproducible comparison, all learning-based methods supporting task-specific adaptation are fine-tuned on the same 1000-pair simulated training split. Specifically, DnCNN is trained for 80 epochs using the Adam optimizer (learning rate of $1\times 10^{-3}$; batch size of 16) with 128×128 random patches and data augmentation. For the diffusion-based editors, we employ Low-Rank Adaptation (LoRA) to efficiently adapt the models. We adopt the optimal LoRA configurations recommended for each model: FLUX.1-Kontext-dev and LongCat-Image-Edit are fine-tuned for 20 epochs and 340 steps, respectively, both with a learning rate of $1\times 10^{-4}$ and a LoRA rank of 32 (bf16 mixed precision). The trainable components of PARE, built upon the Step1X-Edit backbone, are fine-tuned for 20 epochs using the AdamW optimizer (learning rate of $1\times 10^{-4}$; LoRA rank of 64; alpha of 32). InstructPix2Pix is evaluated using its publicly released checkpoint without additional fine-tuning. For all instruction-guided methods, the editing prompt is consistently fixed as “Remove the structured artifacts such as artifacts and stray light while preserving the stellar details”. All training and inference experiments are conducted on NVIDIA GeForce RTX 4090 GPUs (NVIDIA Corporation, Santa Clara, CA, USA) with a fixed random seed of 42 to guarantee reproducibility.

All methods are evaluated on the 300-image simulated test split and the 103-image real on-orbit dataset. The attention-based mask extraction and multi-scale fusion stages of PARE are applied with identical settings across both evaluation sets.

#### 3.4.3. Evaluation Metrics

All evaluations are performed on 8-bit grayscale images. Specifically, the ground-truth, input, and restored outputs are represented as unsigned 8-bit integers.

Unlike natural image editing benchmarks that rely on CLIP-based semantic similarity or perceptual metrics trained on photographic data [8], spaceborne optical images require pixel-level radiometric fidelity, which these metrics cannot capture. We therefore adopt two distinct physics-motivated evaluation protocols for the simulated and real datasets, respectively. Throughout this section $I_{\mathrm{artifact}}$ denotes the original artifact-contaminated image; $I_{\mathrm{proc}}$ denotes the output of a given method; and, when available, $I_{\mathrm{clean}}$ denotes the ground-truth artifact-free image. For a binary region (R), we write ${\bar{I}}^{R}=\frac{1}{\left|R\right|}\sum_{p\in R}I\left(p\right)$ for the mean intensity and $\left|\right|I^{R}{\left|\right|}_{2}^{2}=\sum_{p\in R}I{\left(p\right)}^{2}$ for the total squared energy within R.

**Protocol I—Simulated dataset (ground-truth available).** For the simulated dataset, the evaluation uses a two-part metric set based on the known clean image ($I_{\mathrm{clean}}$).

**Part I-a—Artifact removal effectiveness (inside mask):** The Artifact Removal Rate (ARR, %) measures the fraction of artifact energy eliminated relative to the original contamination:

(13) $$\begin{array}{cc} \mathrm{ARR}\; & =\left(1-\frac{E_{\mathrm{proc}}^{M}}{E_{\mathrm{artifact}}^{M}}\right)\times 100\%,\\ E_{\mathrm{proc}}^{M}\; & =\sum_{p\in M}{\left(I_{\mathrm{proc}}\left(p\right)-I_{\mathrm{clean}}\left(p\right)\right)}^{2},\\ E_{\mathrm{artifact}}^{M}\; & =\sum_{p\in M}{\left(I_{\mathrm{artifact}}\left(p\right)-I_{\mathrm{clean}}\left(p\right)\right)}^{2}. \end{array}$$

Here, M denotes the ground-truth artifact mask region. A higher ARR indicates more complete artifact suppression within the target region.

**Part I-b—Non-target region preservation (outside mask):** To evaluate preservation outside the target region, we compute three pixel-level metrics exclusively on $\bar{M}$—namely, the mask-exterior pixels, using $I_{\mathrm{clean}}$ as the reference:

(14) $$\begin{array}{cc} {\mathrm{O-MSE}}_{\mathrm{sim}}\; & =\frac{1}{|\bar{M}|}\sum_{p\in \bar{M}}{\left(I_{\mathrm{proc}}\left(p\right)-I_{\mathrm{clean}}\left(p\right)\right)}^{2},\\ {\mathrm{O-RMSE}}_{\mathrm{sim}}\; & =\sqrt{{\mathrm{O-MSE}}_{\mathrm{sim}}},\\ {\mathrm{O-PSNR}}_{\mathrm{sim}}\; & =10\log_{10}\left(\frac{255^{2}}{{\mathrm{O-MSE}}_{\mathrm{sim}}}\right), \end{array}$$

where 255 denotes the maximum pixel intensity for standard 8-bit images.

We also report the Outer-SSIM (O-SSIM), the structural similarity index [19] computed between $I_{\mathrm{proc}}$ and $I_{\mathrm{clean}}$ within $\bar{M}$. O-RMSE and O-PSNR provide equivalent information through a monotonic logarithmic transform, with a lower O-RMSE and higher O-PSNR indicating better preservation. O-SSIM further captures structural fidelity that is not reflected by pixel-wise error alone. Any deviation from $I_{\mathrm{clean}}$ within $\bar{M}$ is treated as editing-induced radiometric drift because these pixels carry uncontaminated radiometric information.

**Protocol II—Real dataset (no ground truth).** Since $I_{\mathrm{clean}}$ is unavailable, all metrics reference the original artifact-contaminated image ($I_{\mathrm{artifact}}$), quantifying the magnitude and nature of change rather than deviation from a clean target.

**Evaluation mask construction.** We use fixed image-processing rules to generate a method-agnostic evaluation mask ($M_{\mathrm{eval}}$) independently for each real image before evaluating any method. Specifically, we apply Otsu thresholding on the luminance channel, followed by morphological closing with a 7×7 elliptical kernel and hole-filling. The same mask is used for all compared methods. The *protected region* (P) used for outer-region metrics is defined as the complement of the dilated mask:

(15) $$P=\Omega \setminus \mathrm{dilate}\left(M_{\mathrm{eval}},r=15\right).$$

Here, Ω denotes the full image domain and dilation uses a 31×31 elliptical structuring element (r=15 px), excluding ambiguous boundary pixels from fidelity assessment.

**Part II-a—Artifact suppression (inside $M_{\mathrm{eval}}$):** Artifact Mean Reduction (AMR ↑, %) and Artifact Energy Reduction (AER ↑, %) measure the relative decreases in mean intensity and total squared energy within $M_{\mathrm{eval}}$, respectively. Here, the superscript M denotes restriction to $M_{\mathrm{eval}}$ according to the notation above:

(16) $$\begin{array}{cc} \mathrm{AMR}\; & =\frac{{\bar{I}}_{\mathrm{artifact}}^{M}-{\bar{I}}_{\mathrm{proc}}^{M}}{{\bar{I}}_{\mathrm{artifact}}^{M}}\times 100\%,\\ \mathrm{AER}\; & =\frac{{∥I_{\mathrm{artifact}}^{M}∥}_{2}^{2}-{∥I_{\mathrm{proc}}^{M}∥}_{2}^{2}}{{∥I_{\mathrm{artifact}}^{M}∥}_{2}^{2}}\times 100\%. \end{array}$$

Higher AMR and AER values indicate stronger artifact suppression within the evaluation mask.

**Part II-b—Non-target fidelity (inside P):** We compute two pixel-level metrics against $I_{\mathrm{artifact}}$ within P. The first metric is Outer-MAE (O-MAE↓), which measures the mean absolute pixel deviation:

(17) $$\mathrm{O-MAE}=\frac{1}{\left|P\right|}\sum_{p\in P}\left|I_{\mathrm{proc}}\left(p\right)-I_{\mathrm{artifact}}\left(p\right)\right|.$$

The second metric is Outer-SSIM (O-SSIM ↑), the structural similarity index [19] computed between $I_{\mathrm{proc}}$ and $I_{\mathrm{artifact}}$, restricted to P. A method that leaves P unchanged obtains perfect scores on both metrics, regardless of artifact removal performance.

**Part II-c—Background statistics preservation:** Background pixels (B⊂P) are defined by excluding the brightest 0.5% of pixels from P according to the intensity percentile, thereby removing residual stellar point sources and saturated spots. Background Mean Shift (BMS↓) and Background Std Shift (BSS↓) measure absolute changes in background mean and standard deviation between $I_{\mathrm{proc}}$ and $I_{\mathrm{artifact}}$ over B:

(18) $$\begin{array}{cc} \mathrm{BMS} & =\left|{\bar{I}}_{\mathrm{proc}}^{B}-{\bar{I}}_{\mathrm{artifact}}^{B}\right|,\\ \mathrm{BSS} & =\left|\sigma \left(I_{\mathrm{proc}}^{B}\right)-\sigma \left(I_{\mathrm{artifact}}^{B}\right)\right|. \end{array}$$

Here, σ(·) denotes the standard deviation over B. These metrics capture radiometric drift in background estimation for photometric measurements, which directly affects faint-target detection thresholds.

AMR and AER are interpreted jointly with the Part II-b and Part II-c metrics. A method that achieves a high AMR/AER by indiscriminately reducing image intensities may also exhibit elevated O-MAE in non-artifact pixels and disrupted background statistics. This joint interpretation distinguishes genuine restoration from trivial suppression.

#### 3.4.4. Downstream Task Validation Settings

To verify the physical fidelity and scientific applicability beyond conventional pixel-level metrics, we conduct downstream astronomical star detection and photometric validation. As real on-orbit data lack precise star-level ground-truth catalogs, these evaluations are performed on the simulated dataset, which provides complete and reliable star annotations.

We employ the DAOStarFinder algorithm to extract stellar centroids and photometric flux. To strictly validate the physics-preserving property of PARE, evaluation is restricted to stars located outside the artifact mask. Stellar signals within artifact-contaminated regions are severely occluded, and their restoration involves generative uncertainty. Conversely, non-mask regions represent clean astronomical backgrounds that should remain unchanged. Thus, evaluating outside-mask stars provides an unbiased measurement of whether the method distorts scientifically valuable structures.

Detected stars are matched to ground-truth catalogs via nearest-neighbor search with a 3.0-pixel spatial tolerance. Let $S_{\mathrm{GT}}$ and $S_{\mathrm{proc}}$ denote the sets of detected stars in the ground-truth and processed images, restricted to the outside-mask region. A ground-truth star is considered successfully recovered if its nearest neighbor in $S_{\mathrm{proc}}$ falls within this threshold, forming the matched set ($S_{\mathrm{matched}}\subseteq S_{\mathrm{GT}}$). We quantify the scientific utility using three complementary metrics:

Star extraction accuracy quantifies the preservation rate of valid astronomical sources:

(19) $$\mathrm{Star}\;\mathrm{Extraction}\;\mathrm{Accuracy}=\frac{|S_{\mathrm{matched}}|}{|S_{\mathrm{GT}}|}.$$

Centroid error reflects geometric positional consistency as the mean Euclidean distance between matched pairs:

(20) $$\mathrm{Centroid}\;\mathrm{Error}=\frac{1}{|S_{\mathrm{matched}}|}\sum_{i\in S_{\mathrm{matched}}}{∥c_{\mathrm{proc}}^{\left(i\right)}-c_{\mathrm{GT}}^{\left(i\right)}∥}_{2},$$

where $c_{\mathrm{GT}}^{\left(i\right)}$ and $c_{\mathrm{proc}}^{\left(i\right)}$ denote the centroid coordinates of the *i*-th matched star in the ground-truth and processed images, respectively.

Photometric error measures relative flux deviation to evaluate radiometric consistency:

(21) $$\mathrm{Photometric}\;\mathrm{Error}=\frac{1}{|S_{\mathrm{matched}}|}\sum_{i\in S_{\mathrm{matched}}}\frac{\left|F_{\mathrm{proc}}^{\left(i\right)}-F_{\mathrm{GT}}^{\left(i\right)}\right|}{F_{\mathrm{GT}}^{\left(i\right)}}\times 100\%,$$

where $F_{\mathrm{GT}}^{\left(i\right)}$ and $F_{\mathrm{proc}}^{\left(i\right)}$ denote the extracted photometric flux of the *i*-th matched star.

Together, these metrics systematically verify that PARE preserves the structural and radiometric integrity of astronomical scenes for subsequent analysis.

#### 3.4.5. Ablation Settings

To comprehensively analyze the contribution of each component and the rationality of hyperparameter selection in PARE, we conduct ablation studies on the simulated validation set under Protocol I.

First, to evaluate the architectural design, we compare the full PARE configuration with two variants. The first variant, denoted as *direct edit*, removes the attention-derived artifact mask and the Laplacian pyramid fusion module and directly uses $I_{\mathrm{edit}}$ as the output. This setting evaluates the effect of unconstrained generative editing. The second variant, denoted as *alpha blend*, keeps the attention-derived artifact mask but replaces the Laplacian pyramid fusion module with simple pixel-domain alpha blending between the input image and the edited candidate. This setting evaluates whether multi-scale fusion is necessary when the artifact region is already localized. All variants are evaluated using the same ARR, O-RMSE, and O-SSIM metrics as in Protocol I.

Second, to justify the selection of key hyperparameters, we perform a sensitivity analysis on three critical configurations. Since the simulated dataset provides precise ground-truth artifact masks, the attention threshold (p∈[0.15,0.45]), the morphological kernel size ($k_{m}$∈{3,5,7,9,11}) and the dilation kernel size ($k_{d}$∈{2,3,4,5,6}) are evaluated using mask-level segmentation metrics (e.g., precision, recall, and F2 score) against the ground truth to optimize mask completeness and refinement. Conversely, the depth of the Laplacian pyramid (evaluated at {3,4,5} levels) is assessed using the final image restoration metrics (ARR, O-RMSE, and O-SSIM) to determine the optimal frequency decomposition.

Third, to evaluate the robustness of the instruction-guided editing process, we conduct a prompt sensitivity analysis on 20 randomly selected images from the simulated validation set. We design four prompt variants with increasing levels of semantic detail: (1) a *Minimal* prompt (overly concise input), (2) a *Moderate* prompt focusing on basic artifact removal and star preservation, (3) a *Detailed* prompt specifying the target background restoration, and (4) a *Comprehensive* prompt (our default) that explicitly details all core objectives. These variants are evaluated using the same mask-level and image-level metrics to assess how semantic completeness affects restoration performance.

## 4. Results

### 4.1. Quantitative Results on the Simulated Dataset

Table 1 reports quantitative performance on the simulated dataset under Protocol I; full per-method values are given there and are not repeated below.

**Artifact removal effectiveness (ARR).** DnCNN obtains the highest ARR of 99.35% on the simulated dataset. Among multimodal generative editing methods, PARE achieves the highest ARR of 96.40%, outperforming InstructPix2Pix, FLUX.1 Kontext, and LongCat-Image-Edit. Top-hat obtains an ARR of 86.59%, which is lower than that of PARE.

**Non-target region preservation (outer metrics).** Top-hat and DnCNN achieve the strongest outer-region preservation on the simulated dataset, with O-SSIM values of 1.000 and 0.997, respectively. Among multimodal generative editing methods, PARE obtains the best outer-region fidelity, with an O-RMSE of 5.22, an O-PSNR of 34.32 dB, and an O-SSIM of 0.959. LongCat-Image-Edit obtains an O-RMSE of 16.98 and an O-SSIM of 0.578, while InstructPix2Pix and FLUX.1 Kontext show larger outer-region errors. These results indicate that PARE reduces non-target-region disturbance more effectively than the direct multimodal generative editing baselines.

**Overall observation.** Although DnCNN achieves the highest raw ARR and Top-hat shows the best outer-region preservation, PARE provides the strongest overall performance among multimodal generative editing methods. It combines high artifact removal effectiveness with substantially lower outer-region disturbance, indicating a better balance between artifact removal and non-target-region preservation within this method category.

### 4.2. Quantitative Results on the Real Dataset

Table 2 reports quantitative performance on the real on-orbit dataset under Protocol II. Full per-method values are provided in the table and are not repeated below.

**Artifact suppression (AMR and AER).** DnCNN obtains the highest raw suppression scores on the real dataset, with an AMR of 98.95% and an AER of 99.77%. Top-hat ranks second, with an AMR of 95.20% and an AER of 99.06%. Among multimodal generative editing methods, PARE achieves the strongest artifact suppression, with an AMR of 92.95% and an AER of 98.57%, outperforming InstructPix2Pix, FLUX.1 Kontext, and LongCat-Image-Edit.

**Non-target fidelity (O-MAE and O-SSIM) and background statistics (BMS and BSS).** PARE achieves the best result on all four preservation metrics, with an O-MAE of 0.54, an O-SSIM of 0.997, a BMS of 0.50, and a BSS of 0.70. For O-MAE, BMS, and BSS, the values of PARE are more than an order of magnitude lower than the next-best values. In comparison, Top-hat and DnCNN show larger non-target-region changes and background-statistics shifts, despite their higher raw suppression scores. The remaining multimodal generative editing baselines also show higher O-MAE, BMS, and BSS values than PARE.

**Overall observation.** PARE does not achieve the highest raw artifact suppression, trailing DnCNN and Top-hat by moderate margins. This gap primarily arises because global suppression methods tend to achieve higher raw scores through aggressive smoothing, which inevitably compromises the radiometric fidelity of non-target regions. However, PARE achieves the best outer-region fidelity and background-statistics preservation among all compared methods, including the two strongest suppressors. These results show that high AMR and AER values alone are insufficient for scientific image restoration. By enforcing an explicit spatial constraint, PARE confines artifact suppression to the target region and provides the intended trade-off between artifact removal and radiometric fidelity.

### 4.3. Results of Downstream Task Validation

Table 3 reports the evaluation metrics for stars located outside the artifact mask. The results highlight a significant performance gap between PARE and the baselines, underscoring its scientific utility.

PARE achieves a star extraction accuracy of 99.36% and a sub-pixel centroid error of 0.068 px. These values are virtually identical to the artifact-contaminated input (99.68% and 0.056 px), confirming that our method preserves the structural integrity of non-target regions without data loss. Conversely, all baselines suffer severe performance degradation, with extraction accuracies falling below 22% and centroid errors exceeding 1.8 px. This indicates that their suppression mechanisms distort background structures, leading to massive star loss and unacceptable positional biases.

Regarding photometric consistency, although the input image retains high extraction accuracy, its Photometric Error reaches 49.18% due to flux inflation caused by high-intensity artifacts. By eliminating this radiometric contamination, PARE reduces the error to 17.67% (a 64% improvement). In comparison, baseline methods exhibit even larger deviations (mostly >90%), failing to restore valid radiometric data. Overall, these results demonstrate that PARE effectively balances artifact removal with the preservation of astronomical data fidelity, ensuring reliability for downstream analysis.

### 4.4. Ablation Results

Table 4 reports the ablation results on the simulated validation set. The direct-edit variant achieves a high ARR but introduces substantial outer-region disturbance, as reflected by a much higher O-RMSE and lower O-SSIM. The alpha-blend variant improves outer-region fidelity by using the attention-derived artifact mask, but it remains slightly inferior to the full PARE configuration. The full model achieves the best overall balance between artifact removal and outer-region preservation, confirming the complementary roles of attention-based localization and multi-scale fusion.

**Figure 4:**
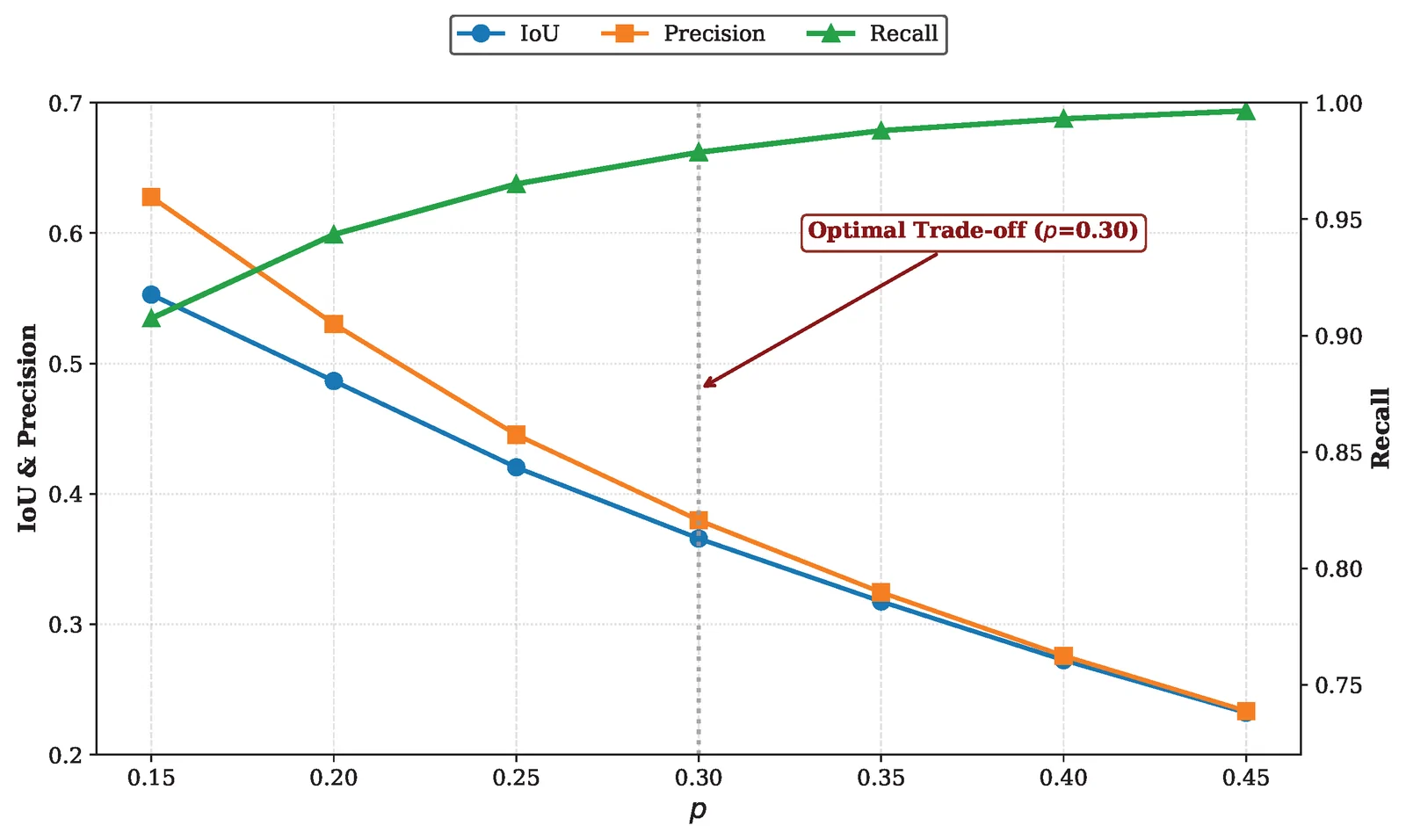
Sensitivity analysis of the attention threshold (*p*) on the validation set. The plot illustrates the trade-off between precision and recall, with p=0.30 selected as the optimal trade-off point to balance mask completeness and background preservation.

**Figure 5:**
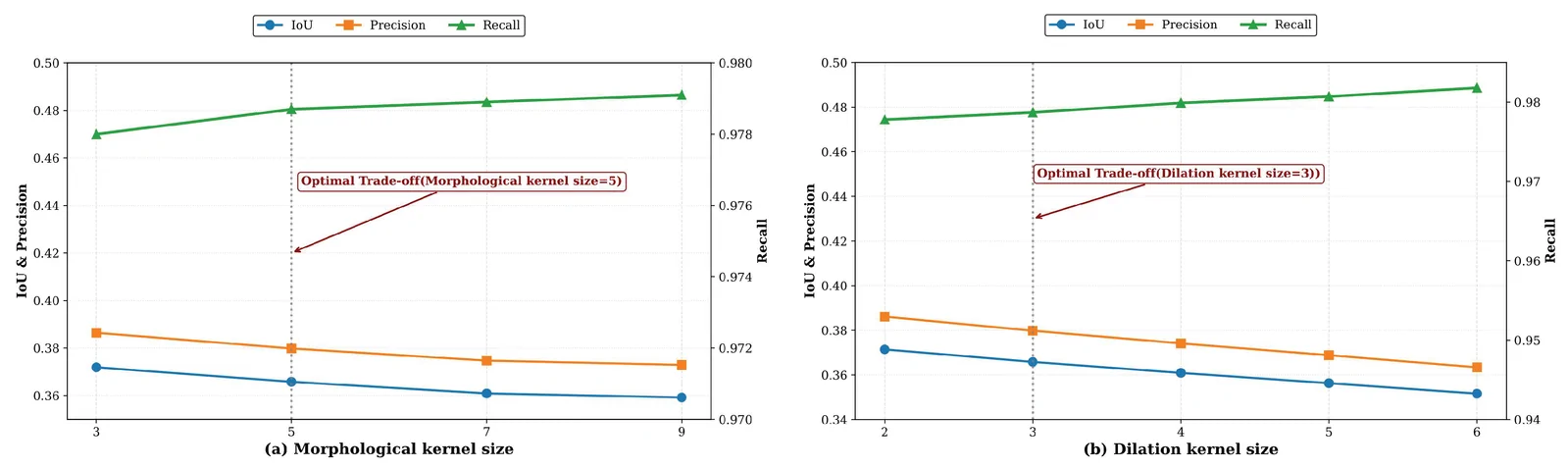
Sensitivity analysis of mask refinement parameters on the validation set. The morphological kernel size (**a**) and dilation kernel size (**b**) exhibit a similar trade-off: increasing the parameter improves mask completeness (recall) but causes continuous degradation in precision and IoU due to mask over-expansion. The selected values (morphological kernel size = 5 and dilation kernel size = 3) achieve the optimal balance.

Furthermore, we evaluate the sensitivity of key hyperparameters to justify their selection. For the attention threshold (*p*) and the morphological kernel size, we utilize mask-level metrics (precision, recall, and IoU) against the ground-truth masks. Figure 4 shows that varying *p* from 0.15 to 0.45 reveals a clear trade-off: higher thresholds increase recall but severely degrade precision. We select p=0.30 as the optimal trade-off, which maintains a high recall of 0.979 while preserving a reasonable precision of 0.380, ensuring comprehensive artifact coverage without excessive background erosion. Similarly, as shown in Figure 5, increasing either $k_{m}$ or $k_{d}$ steadily improves recall by more aggressively filling internal holes and connecting fragmented regions. However, excessively large parameters lead to mask over-expansion, causing a continuous drop in precision and IoU. We select $k_{m}=5$ and $k_{d}=3$ as the optimal trade-offs. At these values, the recall reaches 0.9787, providing a notable improvement over smaller parameters (e.g., 0.9780 for $k_{m}=3$ and 0.9778 for $k_{d}=2$) while avoiding the more significant precision and IoU degradation observed at larger values (e.g., $k_{m}\geq 7$ or $k_{d}\geq 4$).

**Figure 6:**
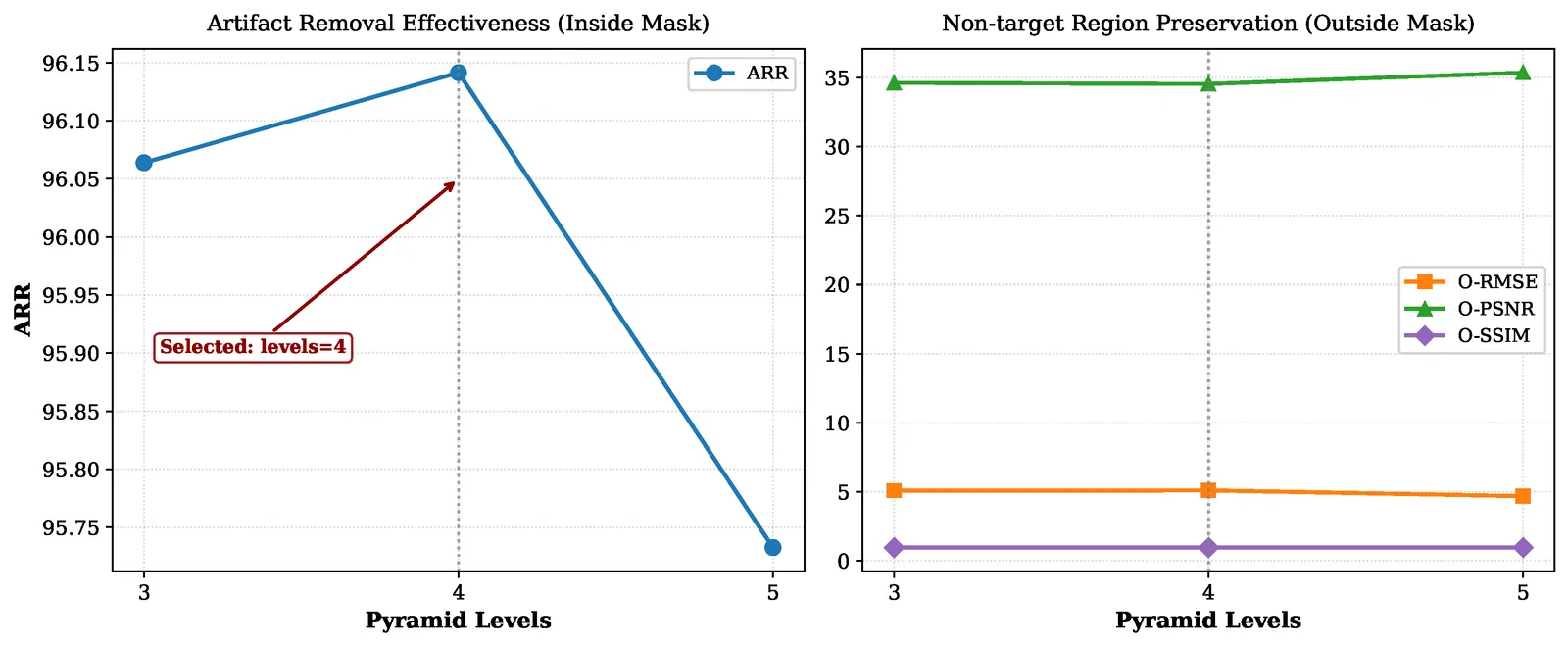
Evaluation of the Laplacian pyramid depth on the validation set. The left plot shows the Artifact Removal Effectiveness (ARR) inside the mask, and the right plot shows the Non-target Region Preservation (O-RMSE, O-PSNR, and O-SSIM) outside the mask. A depth of 4 is selected as the optimal frequency decomposition level.

For the Laplacian pyramid depth, we assess the final image restoration metrics. Figure 6 demonstrates that a depth of 3 is insufficient to separate high-frequency stellar details from low-frequency artifacts (lower ARR), whereas a depth of 5 introduces redundant computations and slightly degrades the outer-region preservation. Consequently, a depth of 4 achieves the highest artifact removal effectiveness inside the mask (ARR = 96.14%) and optimal non-target region preservation outside the mask, confirming it as the optimal frequency decomposition level.

As shown in Table 5, the Minimal prompt yields significantly degraded and highly unstable performance (ARR drops to 70.49%, with a massive Std of 34.49%), indicating that the model fails to interpret the task intent without clear semantic guidance. However, introducing explicit semantic constraints triggers a substantial performance leap. The Moderate prompt achieves an ARR of 97.07%, with a drastically reduced Std (1.37%), demonstrating that clear instructions effectively guide the attention mechanism.

Crucially, when the prompt is refined to comprehensively cover all three core task elements (artifact removal, background restoration, and star preservation), the performance saturates. The *Detailed* and *Comprehensive* variants achieve nearly identical metrics (e.g., ARR ≈ 97.2% and O-SSIM ≈ 0.96), with comparable low variances. This saturation confirms that PARE exhibits high robustness to minor phrasing variations; as long as the prompt semantically covers the essential restoration objectives, the specific wording does not significantly affect the outcome.

### 4.5. Qualitative Analysis

**Figure 7:**
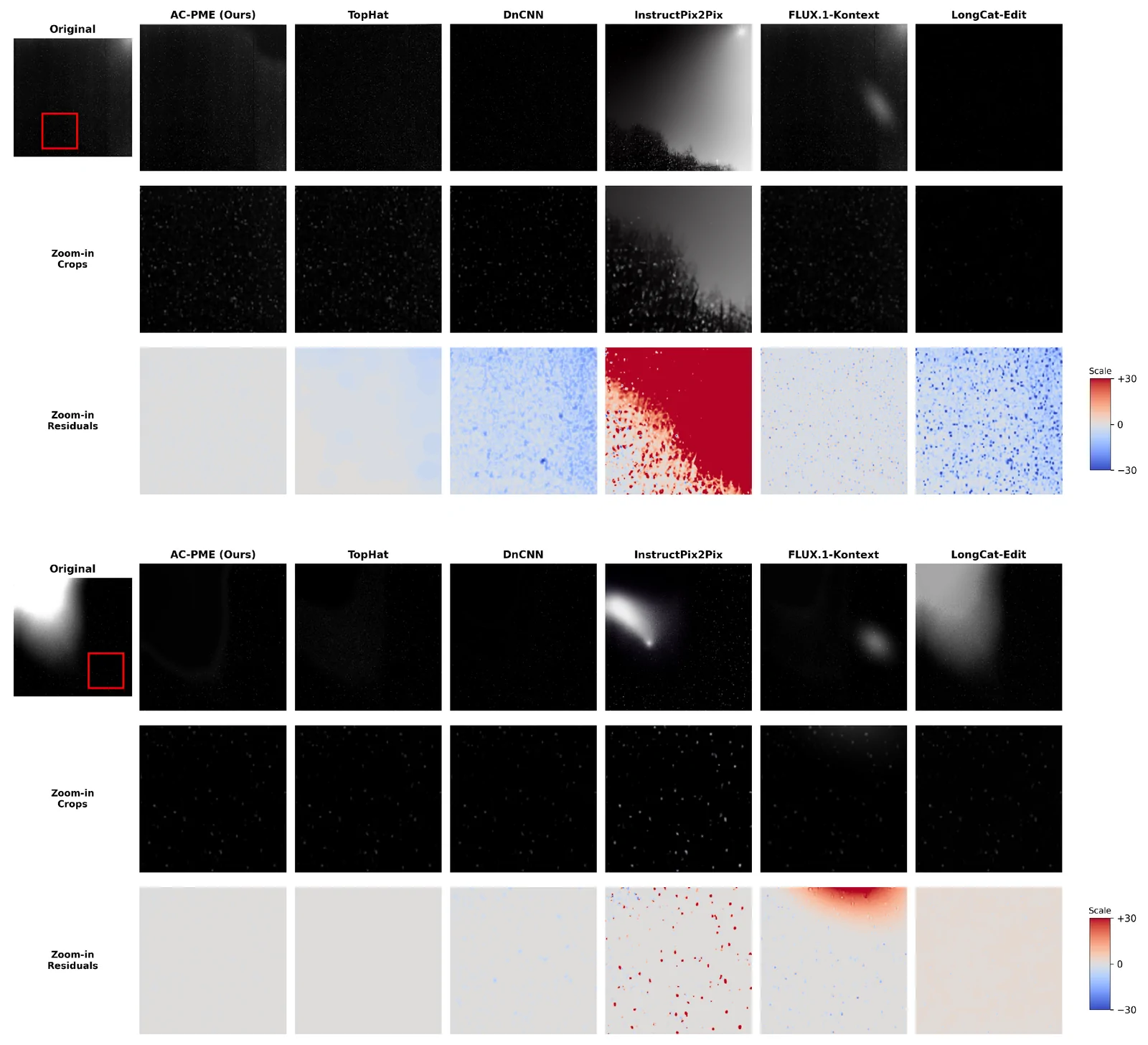
Detailed qualitative comparison on real spaceborne optical images. For each sample, the rows display the full images, zoom-in crops of the artifact regions (marked by red boxes), and the corresponding zoom-in residual maps. The residual maps use a unified linear scale (indicated by the color bar), where blue and red indicate intensity reduction and increase relative to the input, respectively, while white and gray indicate minimal change.

As shown in Figure 7, our method achieves highly localized artifact suppression. While instruction-guided baselines like InstructPix2Pix and FLUX.1 Kontext introduce severe structural distortions (evident from large red/blue residuals) and traditional methods like Top-hat and DnCNN produce broad suppression regions that blur background details, PARE precisely targets the artifacts. Crucially, the residual maps—evaluated under a unified linear scale—reveal that PARE leaves large non-target areas virtually unchanged (white/gray), demonstrating superior background and stellar detail preservation. These visual findings align perfectly with the quantitative metrics in Table 2.

## 5. Discussion

### 5.1. Suppression Strength Versus Radiometric Fidelity

The experimental results reveal an important distinction between artifact suppression strength and radiometric fidelity. In scientific imaging, stronger intensity reduction within the artifact region does not necessarily indicate better restoration quality. This distinction is particularly evident on the real on-orbit dataset (Table 2), where DnCNN and Top-hat obtain higher raw AMR and AER values than PARE but also cause larger changes in non-target regions and background statistics. These results indicate that AMR and AER should not be interpreted in isolation. For spaceborne optical images, restoration quality depends not only on the amount of artifact energy removed but also on whether radiometric information outside the intended artifact region is preserved. The proposed evaluation protocol therefore provides a more appropriate assessment by jointly considering artifact suppression, outer-region fidelity, and background-statistics preservation.

### 5.2. Scientific Utility and Downstream Task Reliability

Beyond pixel-level quality, the practical utility of astronomical restoration lies in its downstream scientific reliability. Quantitative evaluations show that unprocessed images maintain high star detection recall but suffer severe photometric biases from artifact-induced flux inflation. Conversely, conventional and generative baselines frequently eliminate valid stars and introduce positional deviations in non-target regions. PARE effectively resolves this trade-off. By constraining generative modifications to artifact regions and employing multi-scale physics-preserving fusion, PARE achieves near-complete star preservation, sub-pixel centroid stability for outside-mask stars, and substantially reduced photometric errors. This confirms that PARE rigorously preserves the structural and radiometric fidelity essential for trustworthy astronomical measurements.

### 5.3. Generalization from Simulation to Real Observations

The comparison between simulated and real datasets (Table 1 and Table 2) further shows the limited generalization ability of purely supervised restoration under distribution shift. DnCNN achieves strong performance on the simulated dataset because it is trained with paired simulated samples whose artifact distribution is close to that of the test set. This result reflects the advantage of in-distribution supervised learning. However, real on-orbit images contain more complex artifact morphologies, sensor-dependent noise, nonuniform illumination, and background statistics that are difficult to reproduce fully in simulation. As a result, the strong performance of DnCNN on simulated data does not directly imply stable radiometric preservation on real observations. This finding suggests that explicit spatial and radiometric constraints are important when restoration methods are deployed on scientific images whose degradation distribution is not fully known in advance.

### 5.4. Dependence on Editing Quality and Prompt Design

Although PARE incorporates generatively edited candidates for low-frequency replacement, our architecture substantially relaxes the strict requirement for pixel-level editing perfection via two intrinsic mechanisms.

First, dual spatial and frequency constraints inherently filter generative artifacts. The attention-guided mask strictly confines modifications to distorted regions, preventing unintended background alterations. Furthermore, the multi-scale Laplacian fusion extracts only low-frequency corrective information from the candidate while fully preserving high-frequency stellar structures from the original input. Consequently, high-frequency hallucinations (e.g., deformed stars) are automatically excluded during reconstruction.

Second, the framework exhibits strong robustness against diverse prompt formulations. As validated in our prompt robustness analysis (Table 5), PARE does not rely on meticulous prompt engineering. Provided the textual instruction semantically covers the core objectives, the restoration performance remains stable and saturated.

Collectively, these designs decouple the final restoration quality from the pixel-level fidelity of the generative candidate. Instead of requiring flawless outputs, PARE solely demands a semantically correct “low-frequency suppression prior” from the editing branch, ensuring robust restoration against both prompt variations and inherent generative imperfections.

### 5.5. Risks of Direct Generative Editing

The results also reveal the risk of directly applying instruction-driven generative editing to scientific image restoration. Multimodal editing models provide useful semantic priors and can localize visually recognizable artifact patterns through internal attention responses. However, when the generated image is used directly as the restoration output, visually plausible changes may also be introduced outside the artifact region. Such changes may be acceptable in natural image editing, where perceptual quality is often the primary objective, but they can become measurable radiometric errors in spaceborne optical images. The residual visualizations in Figure 7 show that direct generative editing may introduce hallucinated bright structures or modify large non-target areas, which is consistent with the observed degradation in outer-region metrics. These findings support the design choice of using the edited image as a candidate source rather than as the final restored image.

### 5.6. Roles of Attention Masking and Multi-Scale Fusion

The ablation results in Table 4 further clarify the complementary roles of the two main components of PARE. The attention-derived artifact mask determines where generative modification is allowed. Removing this mask leads to substantial outer-region disturbance because the generated output is no longer spatially constrained. The Laplacian pyramid fusion module determines how the edited and observed images are combined. Simple pixel-domain alpha blending under the same mask can preserve much of the outer-region fidelity, but it is more likely to introduce visible boundary artifacts near the mask edge. In contrast, multi-scale fusion allows edited low-frequency content to be introduced within the artifact region while preserving high-frequency stellar structures from the observed image. These results indicate that attention masking and multi-scale fusion address different aspects of editing-induced radiometric drift and are both necessary for achieving a stable balance between artifact suppression and radiometric fidelity.

### 5.7. Computational Overhead and Efficiency Analysis

To provide a comprehensive evaluation, we report the computational overhead (inference time and peak GPU memory) of all compared methods in Table 6. All generative experiments are conducted on a single NVIDIA GeForce RTX 4090 GPU (NVIDIA Corporation, Santa Clara, CA, USA).

As shown in Table 6, traditional and CNN-based methods are extremely fast but often struggle to preserve scientific fidelity. Among generative methods, PARE incurs a higher computational overhead compared to direct editing baselines. This latency gap primarily stems from the architectural difference: while baselines typically rely on a single-stage generation process, PARE employs a multi-stage serial pipeline to strictly enforce physics-preserving constraints, which directly enables our superior scientific utility. Furthermore, the reported inference time explicitly includes the attention-storage overhead required for the saving of intermediate attention maps.

Despite the longer runtime, we emphasize two key aspects regarding PARE’s efficiency in astronomical observations. The first is memory efficiency. PARE requires 16.22 GB of peak GPU memory, which is lower than that required by FLUX.1 Kontext and LongCat-Image-Edit, demonstrating better hardware accessibility. Second is offline scientific application. Spaceborne image restoration is typically an offline post-processing task where radiometric fidelity is paramount, while real-time inference is not strictly required. Furthermore, the current inference time is based on an unoptimized PyTorch 2.5.1 (CUDA 12.1) implementation, and future integration of diffusion acceleration techniques could substantially reduce the runtime.

### 5.8. Limitations and Future Work

Several limitations remain. First, clean references are unavailable for real on-orbit images, so the real-data evaluation relies on input-referenced metrics for non-target fidelity and background-statistics preservation. Consequently, these metrics inherently quantify relative changes with respect to the contaminated input rather than verified absolute restoration accuracy. Although these metrics strictly enforce the constraint that non-artifact regions should remain unchanged, they cannot definitively prove that the artifacts within the masked region are restored to their true, unobservable physical states.

**Figure 8:**
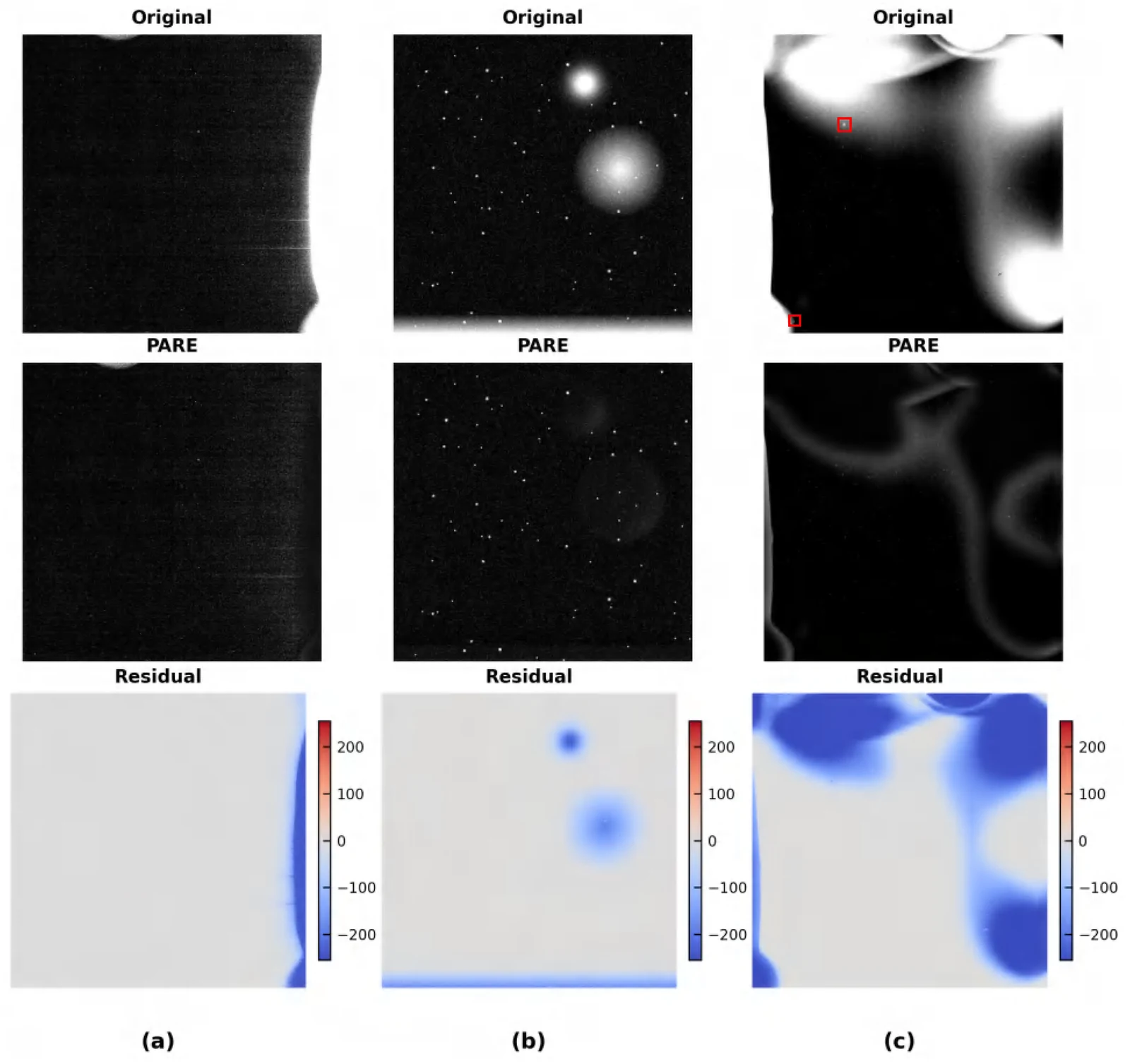
Categorized qualitative comparison on typical and challenging spaceborne optical images. The columns showcase different scenarios: (**a**) typical stray light and halos; (**b**) mixed-intensity morphological stray light and halos; and (**c**) a challenging case featuring severe overlap between sharp-boundary, high-intensity spot halos, stray-light halos, and intricate stellar structures. For each group, the rows display the original contaminated image, the restored result by PARE, and the corresponding residual map (PARE − Original). The residual maps use a unified linear scale (indicated by the color bar on the right of each column), where blue and red denote intensity reduction and increase relative to the input, respectively.

Second, the artifact mask is derived from cross-attention responses and threshold-based refinement. While this annotation-free design improves practicality, mask quality may degrade in extreme scenarios. As visualized in Figure 8, PARE achieves near-perfect artifact suppression and stellar preservation in typical (a) and mixed-intensity (b) scenarios, with residuals strictly confined to the artifact regions. However, when facing severe, large-area contamination where high-intensity artifacts heavily overlap with stellar structures (c), two types of limitations emerge: (1) faint residual traces may persist because the attention mechanism struggles to perfectly disentangle low-frequency artifact halos from high-frequency stellar signals in highly entangled regions, and (2) bright stellar cores within the artifact region (highlighted by the red box in the original image) may be inadvertently removed along with the artifact. Fundamentally, the frequency separation assumption underlying our Laplacian fusion is primarily valid for diffuse, smoothly varying halos; extreme high-frequency structures such as saturated stellar cores or sharp artifact boundaries represent the theoretical performance limit of this hypothesis. By extension, artifacts with fundamentally different physical characteristics, such as cosmic rays (isolated high-frequency outliers) and banding patterns (systematic global striping), also fall outside the processing scope of this frequency-separation framework and are therefore not explicitly evaluated in this study. Collectively, these scenarios define the performance boundary of the current annotation-free masking strategy.

Third, PARE constrains the use of generative outputs during fusion but does not modify the training objective of the underlying diffusion model.

Future work will address these limitations by developing more robust attention aggregation strategies to handle extreme or highly entangled degradation patterns. Additionally, to overcome the computational overhead discussed in Section 5, we plan to integrate computational optimizations, including model quantization, parallel acceleration, and diffusion distillation, to substantially reduce inference latency and memory overhead for practical deployment.

## 6. Conclusions

This paper identified the *editing-induced radiometric drift* problem in diffusion-based image editing for spaceborne optical images. Although instruction-driven generative models provide useful semantic priors for artifact removal, directly using their edited outputs can introduce unintended radiometric changes in non-target regions. To address this issue, we proposed PARE, a physics-preserving attention-guided artifact removal framework that uses MM-DiT I2T cross-attention responses to derive artifact-region masks and combines the edited candidate with the original observation through multi-scale Laplacian pyramid fusion.

Experiments on simulated and real spaceborne optical image datasets show that PARE achieves effective artifact suppression while preserving radiometric information outside the target artifact region. In particular, the results on real on-orbit images demonstrate that PARE does not simply maximize raw intensity suppression; instead, it provides a favorable balance between artifact removal, non-target-region fidelity, and background-statistics preservation. These findings show that explicit spatial constraints and physics-preserving fusion are important for applying generative editing models to scientific imaging tasks that require pixel-level radiometric consistency.

Future work will focus on improving mask robustness, extending the framework to more diverse structured artifacts, and incorporating radiometric constraints into generative model training.

## Figures and Tables

**Table 1:** Quantitative comparison on the simulated test dataset (Protocol I, ground-truth referenced). Results are reported as Mean ± Std to reflect performance variability.

Method	Cat.	ARR ↑ (%)	O-RMSE ↓	O-PSNR ↑ (dB)	O-SSIM ↑
Top-hat [3]	Morph.	86.59 ± 12.77	0.17 ± 0.18	67.46 ± 8.40	1.0000 ± 0.0001
DnCNN [6]	CNN	99.35 ± 0.60	0.44 ± 0.35	59.02 ± 8.37	0.9974 ± 0.0054
InstructPix2Pix [8]	MM Edit	66.17 ± 40.32	47.61 ± 37.53	17.63 ± 7.57	0.4294 ± 0.3016
FLUX.1 [9]		53.79 ± 43.98	22.48 ± 6.89	21.62 ± 3.41	0.7570 ± 0.1369
LongCat [18]		92.06 ± 17.39	16.98 ± 6.57	24.19 ± 3.55	0.5780 ± 0.3156
PARE (Ours)		96.40 ± 2.04	5.22 ± 1.73	34.32 ± 3.19	0.9594 ± 0.0163

↑ indicates that a higher value is better, and ↓ indicates that a lower value is better. Cat., method category; Morph. denotes morphological filtering, CNN denotes CNN-based restoration, and MM Edit denotes multimodal generative editing. LongCat refers to LongCat-Image-Edit, and FLUX.1 refers to FLUX.1 Kontext.

**Table 2:** Quantitative comparison on the real on-orbit dataset (Protocol II, $I_{\mathrm{artifact}}$ referenced.). Results are reported as Mean ± Std to reflect performance variability.

Method	Cat.	AMR ↑ (%)	AER ↑ (%)	O-MAE ↓	O-SSIM ↑	BMS ↓	BSS ↓
Top-hat [3]	Morph.	95.20 ± 5.14	99.06 ± 1.40	8.31 ± 18.12	0.911 ± 0.095	8.04 ± 18.02	10.60 ± 13.31
DnCNN [6]	CNN	98.95 ± 1.21	99.77 ± 0.38	11.73 ± 20.12	0.747 ± 0.221	11.41 ± 20.00	13.25 ± 14.31
InstructPix2Pix [8]	MM Edit	31.16 ± 64.83	17.37 ± 171.18	32.24 ± 34.37	0.656 ± 0.270	29.39 ± 34.72	24.84 ± 22.51
FLUX.1 Kontext [9]		81.96 ± 29.52	88.63 ± 27.46	11.29 ± 19.49	0.848 ± 0.112	7.45 ± 19.38	10.48 ± 10.15
LongCat-Image-Edit [18]		86.26 ± 27.13	88.84 ± 27.08	26.80 ± 43.68	0.597 ± 0.249	24.64 ± 43.96	20.30 ± 21.21
PARE (Ours)		92.95 ± 5.23	98.57 ± 1.45	0.54 ± 1.13	0.997 ± 0.002	0.50 ± 1.10	0.70 ± 0.72

**Table 3:** Downstream validation on star extraction and photometry (evaluated exclusively on stars outside the artifact mask).

Method	Star Extraction Accuracy ↑ (%)	Photometric Error ↓ (%)	Centroid Error ↓ (px)
Input (w/ artifact)	99.68	49.18	0.056
TopHat [3]	19.52	96.14	1.883
DnCNN [6]	0.81	51.29	1.961
InstructPix2Pix [8]	10.24	95.78	1.896
FLUX.1 Kontext [9]	21.43	106.81	1.960
LongCat-Image-Edit [18]	1.71	92.54	2.019
PARE (Ours)	99.36	17.67	0.068

Input (w/ artifact) refers to the original observation containing structured artifacts, without any restoration processing applied.

**Table 4:** Ablation study on the simulated validation set. Results are reported as Mean ± Std to reflect performance variability.

Configuration	ARR ↑ (%)	O-RMSE ↓	O-SSIM ↑
Direct edit (w/o mask, w/o pyramid)	96.17 ± 2.04	22.33 ± 5.61	0.624 ± 0.137
Alpha blend (w/ mask, w/o pyramid)	95.32 ± 1.70	5.90 ± 1.74	0.954 ± 0.015
PARE (full)	95.32 ± 1.60	5.74 ± 1.67	0.956 ± 0.016

**Table 5:** Prompt robustness analysis on 20 randomly selected simulated images from the validation set. Results are reported as Mean ± Std. The four prompt variants are: (1) Minimal: “Remove the artifacts.”; (2) Moderate: “Clean scattered light and artifacts without losing the sharpness and flux of stars.”; (3) Detailed: “Restore a clean dark background by eliminating artifacts and scattered light, while preserving star sharpness and flux.”; and (4) Comprehensive: “Remove the artifacts and scattered light in the image, restore a clean dark background, and preserve the sharpness and flux of stars.”

Prompt Variant	ARR ↑ (%)	O-RMSE ↓	O-PSNR ↑ (dB)	O-SSIM ↑
Minimal	70.49 ± 34.49	4.998 ± 1.255	34.43 ± 2.19	0.952 ± 0.018
Moderate	97.07 ± 1.37	4.709 ± 1.250	34.98 ± 2.32	0.956 ± 0.016
Detailed	97.23 ± 1.31	4.543 ± 1.312	35.33 ± 2.44	0.959 ± 0.017
Comprehensive (Ours)	97.20 ± 1.39	4.550 ± 1.246	35.32 ± 2.50	0.961 ± 0.014

**Table 6:** Computational overhead comparison on the simulated test dataset.

Method	Inference Time (s)	Peak GPU Memory (GB)
Top-hat [3]	0.006	0.00
DnCNN [6]	0.009	0.14
InstructPix2Pix [8]	7.65	15.16
FLUX.1 Kontext [9]	9.80	31.98
LongCat-Image-Edit [18]	67.68	17.18
PARE (Ours)	468.48	16.22

Top-hat and DnCNN are executed on CPU, making direct speed comparisons with GPU-based generative methods inappropriate. The reported time for PARE represents the end-to-end inference, including generative synthesis, attention extraction, multi-scale fusion, and the associated attention storage overhead.

## Data Availability

The original data presented in the study are openly available on GitHub at https://github.com/csx-946/PARE (accessed on 28 August 2026).
